# Supplementary material for: Arithmetic optimization based MPPT for photovoltaic systems operating under nonuniform situations
Source: PLoS One. 2024 Dec 30;19(12):e0311177. doi: 10.1371/journal.pone.0311177 (PMC11684703; doi:10.1371/journal.pone.0311177)
Supplement: Attachment — Submitted filename: Response to Reviewer(s) comments.docx [file pone.0311177.s001.docx]

Response to Reviewer(s)' Comments

We would like to sincerely thank all the reviewers for their comments and suggestions, which help us to enhance our paper greatly. All the comments and suggestions from the reviewers have been considered and responded to carefully. The main changes carried out in the original submission according to the suggestions and comments of the reviewers are shown in **blue** in the revised version. The detailed responses to the reviewers are given as follows.

**Reviewer #1:** - For some reasons, Figures are not included in the final PDF file. So, I am unable to comment on results.

Comment 1: The work is limited to simulation. Mention, to which extent the designed optimization scheme is deployable on a real PV system? What are the challenges involved when the control law is to be implemented on a real system?

**Response 1: The maximum power point is not oscillated around by metaheuristic-based MPPT tactics, in contrast to traditional MPPT techniques like P&O. The computational behaviour of metaheuristic optimisation algorithms is greatly influenced by their control settings. Depending on the nature of the situation, different control parameter values are appropriate. It is necessary to determine the appropriate control parameter values for every optimisation task. It has been discovered by experience that a lengthy process of trial and error is necessary to identify the ideal combination for maximum performance. However, just two parameters need to be adjusted for AOA. Programming requires less work overall. It is anticipated that the conventional microcontroller will provide easy implementation of the algorithm.**

Comment 2: Update the literature review by including notable works on MPPT control such as 10.3390/en16135039, 10.1371/journal.pone.0260480 and 10.3390/app12062773.

**Response 2: Thank you for pointing this out. Changes have been made in the Introduction section. The details of the related reference papers are included in the reference section of the revised manuscript.**

Comment 3: Please write quantitatively especially in the Abstract: How much 'quick' is quick response? How much 'excellent' is excellent?

**Response 3: Thank you for pointing this out. Authors agree with this comment and the abstract has been modified in the revised paper.**

Comment 4: Include more rigorous analysis based on ITAE, IAE, ISE etc.

**Response 4: The comments of the Reviewer are taken into account with gratitude to improve the quality of the manuscript with more sufficient result analysis.**

| **Condition** | **ITAE** | **IAE** | **ISE** |
| --- | --- | --- | --- |
| NUC1 | 1.6006e+06 | 1.9773e+07 | 5.0989e+09 |
| NUC2 | 4.2266e+06 | 2.8296e+07 | 5.6235e+09 |
| NUC3 | 2.5880e+06 | 2.4868e+07 | 6.5006e+09 |

Comment 5: Explicitly mention novelty of the proposed optimization technique.

**Response 5: Thank you for your comment. It is a major problem to correctly track the GMPP in extreme weather circumstances, and traditional MPPT approaches are likely to be caught in between LMPPs and ineffectual. Using optimisation algorithms is a useful strategy for tracking GMPP in non-uniform solar irradiation conditions. Therefore, AOA-MPPT method under uniform and non-uniform conditions are investigated in this paper**. **In this method, the oscillations at steady state are reduced, which reduces the power loss. It requires less time to converge.**

Comment 6: Avoid referring to waveforms/curves in a graph from their color. e.g. The 'green dotted line outlines the ideal ..."

**Response 6: Thank you for your valuable comment. Correction have been carried out in the respective section of the revised manuscript.**

**Reviewer #2:**

Comment 1. Abstract is ambiguous, kindly arrange the abstract which can contain background of the work, objective, method used and results achieved.

Response 1: **Thank you for pointing this out. Authors agree with this comment and the abstract has been modified in the revised paper.**

Comment 2: Try to expand the literature review including some recent works (of last 3-years) in the similar field.

**Response 2: As suggested by the reviewer, the latest literature related to MPPT is included in the Introduction section of the revised manuscript. The details of the related reference papers are included in the reference section of the revised manuscript.**

Comment 3. Figures are not shown in the paper. Only figure numbers are included. Why?

**Response 3: Thank you for your valuable comment. Correction have been carried out in the all sections of the revised manuscript.**

Comment 4. The validation of results is missing. Kindly validate the results either by hardware setup or at least by real-time HIL emulator.

Response 4: **Thank you for your valuable comment. Experimental setup and results included in the revised manuscript.**

Comment 5. Kindly analyze the performance under dynamic conditions.

Response 5: **Thank you for your comment. An effective AOA-MPPT algorithm may quickly and accurately converge to the necessary power, regardless of a gradual or abrupt change in sun irradiation. The maximum power obtained, settling time, and Tracking efficiency of three MPPT approaches are compared and depicted in Figure 15-19. According to the simulation results, it is concluded that the AOA-MPPT outperforms in terms of faster convergence to GP, higher efficiency, and less oscillations. The efficacy of the proposed MPPT method is confirmed for both partial shaded conditions and rapid changing irradiance conditions. Comparative results show that the proposed method exhibits greater performance than other methods under steady state and dynamic conditions.**

Comment 6. Kindly rewrite the conclusion. Only novelty and originality of the work should be included in the conclusion. Kindly add 1-2 lines of future work in the last of conclusion section.

**Response 6:** **Thank you for your comment.** **As suggested, conclusion section has been modified in the revised manuscript.**

Comment 7. References are very old. Kindly cite at least 4 to 5 recent papers to validate that your work is novel and attempted by you first time in this domain.

**Response 7: As suggested by the reviewer, the latest literature related to MPPT is included in the Introduction section of the revised manuscript. The details of the related reference papers are included in the reference section of the revised manuscript.**

Comment 8. Authors have reference [40] in the text but it has not been given in the reference list.

**Response 8: Thank you for pointing this out. Changes have been made in the revised manuscript.**

**Reviewer #3:**

Comment 1. Figures are not incorporated in the manuscript. Plz check.

Response 1: **Thank you for pointing this out. Changes have been made in the revised manuscript.**

Comment 2. Why author is considered sepic converter.

**Response 2: Thank you for your comment. In this paper, SEPIC which has a high efficiency coefficient, has been selected. Even though it's a fourth-order electronic circuit, it has a lot of benefits that make it ideal for photovoltaic applications. These benefits include DC output current, series capacitor isolation between the input and output sides, flexible output gain, and non-inverting DC output voltage. The SEPIC may step up or step down the input voltage and operate in both buck and boost modes. It can thus track the maximum power point of the PV system at both the PV power level and input voltage. Despite having two inductors, the SEPIC converter's input ripple current is decreased, which lowers the peak inductor current and lowers the inductors' losses. These properties make SEPIC converter a suitable candidate for PV applications.**

Comment 3. The abstract should be revised. Some parts of the abstract are unnecessary and should be removed. The main contributions should be highlighted in the abstract. Addressing some quantitative findings compared to available research works in the abstract is suggested.

**Response 3: Thank you for your comment. The findings demonstrate that AOA can follow MPP in 200–300ms under a variety of environmental changes. Moreover, the suggested AOA-MPPT has a tracking efficiency of almost 99% and settling time of 200-300ms. Besides, it has the ability to effectively manage the situation of partial shading. When it comes to tracking capabilities, transient behaviour, and convergence, AOA performs better than both P&O and GWO.**

Comment 4. How were the parameters of the proposed method selected?

**Response 4: Thank you for pointing this out.** **The two parameters are μ and α. The results of this research indicate that whereas α is a sensitive parameter that specifies the exploitation accuracy across the iterations and is fixed at 5, μ is a control parameter that adjusts the search process and is fixed at 0.5.**

Comment 5. Please validate the proposed method using load variation, and disturbances.

Response 5: **Thank you for your comment. As suggested,** **a new figure is included in the results and discussion section of the revised manuscript to describe the effect of load variation on proposed method. When load has been reduced, the maximum power across the load also reduced. The proposed MPPT algorithm effectively handle the load variations.**

**Figure 20. Output power due to load variations**

Comment 6. To show the efficacy of the proposed control method statistical analysis is needed. Please add it.

**Response 6**: **The comments of the Reviewer are taken into account with gratitude to improve the quality of the manuscript with more sufficient result analysis.**

| **Condition** | **ITAE** | **IAE** | **ISE** |
| --- | --- | --- | --- |
| NUC1 | 1.6006e+06 | 1.9773e+07 | 5.0989e+09 |
| NUC2 | 4.2266e+06 | 2.8296e+07 | 5.6235e+09 |
| NUC3 | 2.5880e+06 | 2.4868e+07 | 6.5006e+09 |

Comment 7. Please cite some paper <https://doi.org/10.1002/oca.2798>, 10.1109/JSYST.2020.3020275, [https://doi.org/10.1049/iet-gtd.2018.5019, https://doi.org/10.3390/electronics11060927](https://doi.org/10.1049/iet-gtd.2018.5019,%20https://doi.org/10.3390/electronics11060927) etc <https://doi.org/10.1002/2050-7038.2824> etc.

Response 7: **Thank you for pointing this out. Changes have been made in the Introduction section. The details of the related reference papers are included in the reference section of the revised manuscript.**

Comment 8. Provide proof of the stability and convergence properties of the Proposed algorithm under various assumptions.

**Response 8: Thank you for your comment. An effective AOA-MPPT algorithm may quickly and accurately converge to the necessary power, regardless of a gradual or abrupt change in sun irradiation. The maximum power obtained, settling time, and Tracking efficiency of three MPPT approaches are compared and depicted in Figure 15-17. According to the simulation results, it is concluded that the AOA-MPPT outperforms in terms of faster convergence to GP, higher efficiency, and less oscillations.**

Comment 9. A major issue is the experimental analysis or real time work.

Response 9: **Thank you for your valuable comment. Experimental setup and results included in the revised manuscript.**

Comment 10. The contribution is in general not so highlighted area.

**Response 10: Thank you for your comment. In this paper, AOA-MPPT is proposed to track maximum power from PV under uniform and nonuniform conditions. Because AOA algorithms explore a large search space, AOA lower the likelihood of adhering to local maxima. Furthermore, this is less expensive computationally than AI methods. With minimal steady-state oscillations, the suggested AOA-MPPT technique can track GMPP with extremely high tracking speed and efficiency.**

Comment 11. How the different PSC conditions are arrived at merits further explanation in the text.

**Response 11: Thank you for your comment. Three Non-Uniform Conditions have been chosen in which GMPP is located in middle, end and start positions respectively.**

Comment 12. If possible, Table should include the standard deviation (or variance for that matter) of power tracked after the MPP is settled (in the steady-state) to quantify the amount of oscillations.

Response 12: **Thank you for your comment**. **Once the steady state is reached, the AOA-MPPT algorithm continues to maintain the MPP with almost zero fluctuation, as shown in Figure 10.**

**Figure 10 Maximum power delivered to the load for NUC-1**

Comment 13. Also, the authors applied shading patterns for GP at the middle and at the end of P-V curve, it is better to add a shading pattern case for GP located at the beginning.

**Response 13: Thank you for pointing out.** **As suggested, shading pattern case for GP located at the beginning has been included in the revised manuscript. When it comes to tracking capabilities, transient behaviour, and convergence, AOA performs better than both P&O and GWO. These instances lead to the conclusion that the samples' MPP convergence is mostly independent of their starting positions.**

|   (a) |   (b) |   (c) |
| --- | --- | --- |

**Figure 8. PV characteristics under NUCs with different GP positions**

Comment 14. Conclusions section must be improved, underlining the relevant contributions and results and also future scope.

**Response 14: Thank you for your comment.** **As suggested, conclusion section has been modified in the revised manuscript.**

**Reviewer #4**: There is no figures in the paper. The authors are very careless while uploading the paper. However, the novelty of the paper is also poor. It needs complete update. The results are not properly presented. The analysis is not done properly.

**Response:** **Thank you for your comment. Corrections have been made in the revised manuscript. In this paper, AOA-MPPT is proposed to track maximum power from PV under uniform and nonuniform conditions. Because AOA algorithms explore a large search space, AOA lower the likelihood of adhering to local maxima. Furthermore, this is less expensive computationally than AI methods. With minimal steady-state oscillations, the suggested AOA-MPPT technique can track GMPP with extremely high tracking speed and efficiency. The proposed AOA-MPPT's efficacy under different insolation patterns has been validated using three nonuniform conditions in terms of convergence, tracking speed, steady state oscillations, and tracking efficiency. The tracking efficiency of the AOA-MPPT is above 99% and settling time is 200 to 300ms for all three non-uniform conditions.**

The authors are very grateful to the editor and reviewers for all their careful assessments and constructive suggestions, which have helped to improve the presentation and enhance the quality of the research paper. The above Response sheet is also attached with the revised manuscript.
