# Supplementary material for: Arithmetic optimization based MPPT for photovoltaic systems operating under nonuniform situations
Source: PLoS One. 2024 Dec 30;19(12):e0311177. doi: 10.1371/journal.pone.0311177 (PMC11684703; doi:10.1371/journal.pone.0311177)
Supplement: Attachment — Submitted filename: Response to Reviewer-revision2.docx [file pone.0311177.s002.docx]

Response to Reviewer(s)' Comments

We would like to sincerely thank all the reviewers for their comments and suggestions, which help us to enhance our paper greatly. All the comments and suggestions from the reviewers have been considered and responded to carefully. The main changes carried out in the original submission according to the suggestions and comments of the reviewers are shown in **blue** in the revised version. The detailed responses to the reviewers are given as follows.

**Reviewer #1:** Authors have addressed all the comments suggested. The revised version of the paper has been significantly improved. The paper can be accepted in its present form.

**Response: Thank you for your valuable comment.**

**Reviewer #3:**

**Comment** **1.** So many comments are not answer properly. like Q5, 6 and 8, 12.

**Response 1: The comments of the Reviewer are taken into account with gratitude to improve the quality of the manuscript with more sufficient result analysis. Statistical analysis and experimental results are included in the revised manuscript.**

**Table 3 Numerical Results obtained from AOA and GWO method**

| **Algorithm** | **Condition** | **MAE** | **MSE** | **RMSE** | **Mean** | **SD** | **SD in steady state** |
| --- | --- | --- | --- | --- | --- | --- | --- |
| AOA | NUC1 | 34.5 | 8.9031e+03 | 94.35 | 603.2080 | 0.0459 | 0.0029 |
|  | NUC2 | 108.0709 | 2.1478e+04 | 146.55 | 441.9291 | 0.1368 | 0.0316 |
|  | NUC3 | 28.7572 | 7.5173e+03 | 86.7026 | 711.6504 | 0.0310 | 0.0019 |
| GWO | NUC1 | 45.6 | 9.686e+03 | 98.42 | 599.3480 | 0.052 | 0.0042 |
|  | NUC2 | 120.34 | 2.3311e+04 | 152.68 | 420.5824 | 0.142 | 0.0426 |
|  | NUC3 | 33.26 | 8.619e+03 | 92.84 | 700.7824 | 0.036 | 0.0026 |

The statistical terms such as, mean, standard deviation (SD), mean absolute error (MAE), mean square error (MSE), and root mean square error (RMSE) are used to analyse the results under non uniform conditions, as illustrated in Table 3. In addition, standard deviation of power tracked after the MPP is settled (in the steady-state) is tabulated to quantify the amount of oscillations.

According to Figure 18a, the convergence time with GWO-MPPT for UC is 280ms and it can be reduced to 160ms with the aid of AOA-MPPT. Also, the convergence time is reduced to 120ms with AOA and 150ms with GWO for change in irradiance condition from UC to NUC1.

Figure 20 is included to describe the effect of load variation on proposed method. When resistive load has been reduced from 40Ω to 20Ω, the disturbance occurs in output voltage of converter but the AOA tracks the actual power within 10ms.

**Comment** **2.** Please read some paper for answer these questions. 10.1109/TIA.2024.3413052, and 10.1109/TIA.2023.3321031.

**Response 2: Thank you for pointing this out. Changes have been made in the respective section. The details of the related reference papers are included in the reference section of the revised manuscript.**

**Comment** **3.** Follow these papers how the stability analysis and convergence characteristics are analysis and also cite these.

**Response: Thank you for pointing this out. Changes have been made in the results section. The details of the related reference papers are included in the reference section of the revised manuscript.**

According to Figure 18a, the convergence time with GWO-MPPT for UC is 280ms and it can be reduced to 160ms with the aid of AOA-MPPT. Also, the convergence time is reduced to 120ms with AOA and 150ms with GWO for change in irradiance condition from UC to NUC1.

**Comment** **4.** please take more case studies to analysis and verify the proposed algorithm .

**Response: Thank you for your comment. Three non-uniform insolation conditions with different GP positions and two different fast irradiance varying conditions have been analysed. An effective AOA-MPPT algorithm may quickly and accurately converge to the necessary power, regardless of a gradual or abrupt change in sun irradiation. The maximum power obtained, settling time, and Tracking efficiency of three MPPT approaches are compared and depicted in Figure 15-19.**

**Comment** **5**. The future scope should be broad. not like a comment.

**Response: Thank you for your comment.** **As suggested, conclusion section has been modified with future scope in the revised manuscript.**

In light of these findings, the work's next stage will involve hardware validation of the suggested AOA-MPPT algorithm for PV systems operating in uneven and quickly changing settings. It is anticipated that the PV community, comprising researchers and practitioners alike, will be highly interested in our work. In order to build a solar photovoltaic grid-connected power generating system in the future, authors will incorporate the suggested AOA-MPPT technique with PV inverters, with the goal of enhancing the overall energy harvesting efficiency. Analysis can be done on any single switch DC-DC boost converter with numerous peaks in the P-V curve can use the AOA-MPPT. In addition, the authors will take into account a practical solution to address the issue of partially shadowing the PV array surface.

**Comment** **6.** The statical analysis is not correct.

**Response 6: The comments of the Reviewer are taken into account with gratitude to improve the quality of the manuscript with more sufficient result analysis.**

**Table 3 Numerical Results obtained from AOA and GWO method**

| **Algorithm** | **Condition** | **MAE** | **MSE** | **RMSE** | **Mean** | **SD** | **SD in steady state** |
| --- | --- | --- | --- | --- | --- | --- | --- |
| AOA | NUC1 | 34.5 | 8.9031e+03 | 94.35 | 603.2080 | 0.0459 | 0.0029 |
|  | NUC2 | 108.0709 | 2.1478e+04 | 146.55 | 441.9291 | 0.1368 | 0.0316 |
|  | NUC3 | 28.7572 | 7.5173e+03 | 86.7026 | 711.6504 | 0.0310 | 0.0019 |
| GWO | NUC1 | 45.6 | 9.686e+03 | 98.42 | 599.3480 | 0.052 | 0.0042 |
|  | NUC2 | 120.34 | 2.3311e+04 | 152.68 | 420.5824 | 0.142 | 0.0426 |
|  | NUC3 | 33.26 | 8.619e+03 | 92.84 | 700.7824 | 0.036 | 0.0026 |

The statistical terms such as, mean, standard deviation (SD), mean absolute error (MAE), mean square error (MSE), and root mean square error (RMSE) are used to analyse the results under non uniform conditions, as illustrated in Table 3. In addition, standard deviation of power tracked after the MPP is settled (in the steady-state) is tabulated to quantify the amount of oscillations.

**Comment** **7**. Experimental results are not correct. Please take proper scale and give in paper.

**Response: Thank you for your valuable comment. Experimental setup and results included in the revised manuscript.**

The authors are very grateful to the editor and reviewers for all their careful assessments and constructive suggestions, which have helped to improve the presentation and enhance the quality of the research paper. The above Response sheet is also attached with the revised manuscript.

1. **EXPERIMENTAL RESULTS**

The experimental setup for the proposed system is developed according to the simulation specification as shown in Figure 21. The proposed control algorithm for the DC-DC converter is implemented using PIC 16F877A from Microchip.

Figure 21. Experimental Setup for the Proposed PV system

(a)

(b)

(c)

(d)

Figure 22. Experimental Results (a) Control signal from MPPT controller (b) Converter output under UC

Figure 22 shows the experimental results for proposed solar PV system under UC where the results obtained are similar to the results obtained from simulation. Figure 22a shows the control signal obtained from the proposed AOA-MPPT algorithm to the switches which also shows the voltage across the diode. Figure 22b shows the converter’s steady state output voltage of 198 V and current of 3.74 A when **NUC-3:** Insolation = [1000 600 1000 1000] $W/m^{2}$ and Temperature = 25°C. Figure 22c depicts the variation of voltage and current during non-uniform environmental conditions described in NUC-3. Figure 22.d. depicts the variation of voltage and current during fast changing solar irradiance condition. The experimental results evidences the efficacy of proposed AOA-algorithm under different circumstances and closely matches with simulation results.
